# A Study on the Intervention Effect of the Creative Problem Solving (CPS) Model in Serious Games for Children with Autism Spectrum Disorder

**DOI:** 10.3390/bs16071214

**Published:** 2026-07-17

**Authors:** Huili She, Tianyang Xu, Ruhao Liu, Wenting Jiang, Ruting Shen, Huibo Xu

**Affiliations:** 1 School of Art and Design, Anhui University of Technology, Ma’anshan 243000, China; ysxyshl@ahut.edu.cn (H.S.); xutianyang@ahut.edu.cn (T.X.); jiangysxy@ahut.edu.cn (W.J.); 2 School of Journalism and New Media, Xi’an Jiaotong University, Xi’an 710049, China; 3 Autism Division, Ma’anshan Special Education School, Ma’anshan 243000, China; srt970323@email.swu.edu.cn; 4 College of Art and Design, Nanjing Forestry University, Nanjing 210037, China; xuhuibo@njfu.edu.cn

**Keywords:** autism spectrum disorder (ASD), serious games, creative problem solving (CPS), digital design

## Abstract

Serious games are increasingly used as supportive tools in interventions for children with Autism Spectrum Disorder (ASD). However, many existing game-based interventions are not clearly grounded in established educational or therapeutic models, which may limit their consistency and overall effectiveness, as behavioural science posits that intervention efficacy is closely tied to alignment with evidence-based principles of behavioural shaping and contingent reinforcement. In response, this study introduces the Creative Problem Solving (CPS) model into serious game design and proposes a more structured digital intervention approach that connects game mechanics with intervention theory, a design logic that aligns with behavioural science tenets of scaffolded skill building and incremental goal setting to foster sustainable behavioural change in neurodiverse children. In September 2024, 48 children with mild-to-moderate ASD were recruited from local special education schools. Based on their behavioural characteristics, a series of customised serious games was developed using CPS as the guiding framework. To examine the impact of this model, a 12-week intervention experiment was carried out between March and June 2025. Participants were randomly assigned to an experimental group or a control group. The findings showed that children in the experimental group improved more than those in the control group in four areas: language communication, social interaction, sensory processing, and health-related behaviours. Improvements in language and social performance were especially noticeable. Observational data also suggested that children in the experimental group became more willing to cooperate and participate as the intervention progressed, while non-engaged behaviours decreased considerably during the later stages. Overall, the results indicate that incorporating structured educational models such as CPS into serious game design may help enhance the stability and effectiveness of digital interventions for children with ASD. This study also provides practical evidence for developing more targeted serious games for this population.

## 1. Introduction

Autism spectrum disorder (ASD) is characterised by persistent difficulties in social communication and restricted, repetitive patterns of behaviour (Kangarani-Farahani et al., 2024; Jiang et al., 2024). In the absence of specific pharmacological interventions, educational interventions remain the primary means of improving core symptoms and promoting social functioning (Chung et al., 2024; T. E. Smith et al., 2021; L. Y. Li et al., 2012). With their structured, highly interactive, and replicable learning environments, serious games are increasingly emerging as a promising tool for ASD intervention (Dantas & do Nascimento, 2022). Existing research has reported improvements in areas such as emotion recognition (Alves et al., 2013), attention (Carvalho et al., 2015), and social functioning (Kang & Chang, 2018). However, existing serious games tend to focus on isolated skill training, lacking a coherent, process-oriented theoretical framework to guide their design (Carneiro et al., 2024). A systematic review by Walsh et al. (2024) in the Autism explicitly states that, in this field, ‘there is currently no consensus on how best to design games to maximise learning opportunities’. This fragmented design approach directly leads to diminished intervention outcomes: whilst children with ASD may practise specific social behaviours proficiently in virtual environments (Ramli et al., 2022), their inability to systematically internalise social logic makes it difficult for them to transfer these acquired skills to real-life social situations characterised by multiple steps (Qahmash, 2018), dynamic interactions, and uncertainty. Consequently, there is an urgent need for a game-based intervention that systematically constructs problem-solving processes, rather than merely rehearsing isolated behaviours.

The CPS model provides an ideal design framework to fill this gap. Originating in educational psychology, CPS structures the thinking process into four stages: understanding the challenge, generating ideas, seeking acceptance, and designing and evaluating (Van Hooijdonk et al., 2020; Bagassi & Macchi, 2020). It is important to note that the introduction of CPS in this study is not intended to replace established intervention models such as Applied Behaviour Analysis (ABA) or Key Response Training (PRT) (Roane et al., 2016; L. Wang et al., 2024). As an educational model, CPS organises learning activities into a staged and process-oriented sequence rather than a single behavioural training technique (Van Hooijdonk et al., 2020; Bagassi & Macchi, 2020). In contrast to ABA or PRT, which mainly specify principles for shaping target behaviours through reinforcement and pivotal-response strategies, CPS is used in this study as a design scaffold for translating intervention goals into serious game mechanics. Its value lies in supporting creative thinking, problem decomposition, emotional expression, social negotiation, and practical decision-making (Sun et al., 2022; Hijab et al., 2024; De Angelis et al., 2024). Therefore, CPS was not adopted as a substitute for established ASD intervention approaches, but as a structured design framework that can organise fragmented game tasks into a coherent developmental process. More specifically, each CPS component was theoretically linked to a corresponding ASD-related outcome domain. The “understanding the challenge” stage uses visually explicit stories, predictable task sequences, and clear goal cues to reduce ambiguity, support attentional orientation, and help children regulate responses to sensory and contextual stimuli. The “generating ideas” stage requires children to select, compare, and explain possible solutions, thereby encouraging flexible thinking, intentional language use, and purposeful communication. The “seeking acceptance” stage embeds role allocation, turn-taking, peer negotiation, and joint decision-making, which directly targets social initiation, reciprocity, and cooperative behaviour. Finally, the “designing and evaluating” stage provides immediate feedback, retry opportunities, and positive reinforcement, which are expected to promote self-monitoring, adaptive adjustment, task persistence, and health-related/adaptive behaviour. In this way, the CPS model provides a mechanism for connecting game mechanics with measurable behavioural outcomes, including language communication, social skills, sensory processing, and health-related behaviour.

The strength of the latter lies in its role as a theory of intervention effectiveness, shaping specific behaviours through consequence-based reinforcement. In contrast, CPS functions here as a high-level design process theory for serious games; its sequential stages can be precisely mapped onto game levels, narrative, and interactive logic, providing children with ASD a coherent ‘problem-solving scaffold’ (Thomas & Huffman, 2020). For children with ASD who prefer structured environments (Zhan et al., 2024), this design—which transforms fragmented skill practice into a coherent cognitive journey—is more likely to foster deep, transferable social skills.

However, research on integrating CPS systems into digital interventions for ASD remains scarce (She et al., 2025); although positive effects on collaboration and engagement have been observed (Sun et al., 2022; Hijab et al., 2024), no study has yet translated the complete CPS cycle into a serious game and tested its incremental effects. This gap, characterised by both a ‘theory-to-design translation deficit’ and a ‘lack of empirical research’, serves as the starting point for this study.

To this end, we developed a serious game and intervention protocol based on the CPS model framework. To test this hypothesis, we conducted a 12-week randomised controlled trial, assigning 48 children with mild-to-moderate ASD to either the CPS game group or a conventional game group with a matching duration. The primary research question is: Can CPS-driven design produce significant improvements in the dimensions of verbal communication, social skills, sensory processing, and health behaviours among children with ASD? Methodological questions—namely, how the CPS model is systematically translated into game mechanics—are addressed through a detailed design exposition, which forms the basis of the comparative trial. The core contribution of this study lies in the empirical testing of a paradigm shift in design: a transition from universal technology-enhanced training to a theoretically structured, process-oriented game-based intervention. By presenting both the design translation and behavioural outcomes, it provides a replicable framework and rigorous evidence to advance digital interventions for children with ASD.

## 2. Background and Related Work

Serious games, designed for non-recreational purposes, are increasingly being used in ASD intervention due to their ability to provide a structured, predictable, and repeatable learning environment (Dantas & do Nascimento, 2022; Wilson, 2024). Empirical research indicates that serious games can effectively enhance the cognitive abilities, sensory processing, and social functioning of children with ASD (D. Li et al., 2023; Kang & Chang, 2018; E. Smith et al., 2021). Compared to traditional face-to-face interventions, digital tools can reinforce core instructional stimuli and reduce environmental distractions, thereby alleviating the selective attention overload commonly experienced by children with ASD (Hayes et al., 2018). However, a systematic review by Carneiro et al. (2024) points out that the majority of current serious games for ASD are not explicitly grounded in established intervention theories; their design is often based on intuitive experience, which has prevented the academic community from reaching a consensus on how game mechanics should be optimally configured to maximise therapeutic outcomes.

The CPS model is a structured educational framework that promotes the development of cognitive, social, and emotional skills through four sequential stages: ‘understanding the challenge—generating ideas—seeking acceptance—designing and evaluating’ (Van Hooijdonk et al., 2020; Bagassi & Macchi, 2020; Sha et al., 2025). Originally applied in mainstream education, CPS has in recent years been introduced into ASD intervention. Research indicates that CPS-based approaches can effectively stimulate active participation in children with ASD, enhance their motivation to learn, and promote peer collaboration (She et al., 2025; Hijab et al., 2024; Sun et al., 2022). CPS emphasises structured, step-by-step reasoning, which aligns closely with the characteristic that children with ASD learn more effectively in predictable, clearly structured environments (Zhan et al., 2024). Furthermore, CPS has demonstrated a positive effect on collaborative behaviour and social interaction in both mainstream and special education settings (Sun et al., 2022; De Angelis et al., 2024).

Although serious games and the CPS model each show promising application prospects, research on the systematic integration of the two is virtually non-existent. Existing research either applies CPS to other media such as digital picture books (She et al., 2025), or merely incorporates fragmented pedagogical elements into serious games, lacking a coherent theoretical core. The key gap lies in the fact that the phased cognitive logic of CPS has not yet been systematically translated into core game mechanics, but is merely viewed as a loose pedagogical intervention superimposed upon the game. Carneiro et al. (2024) explicitly highlight this deficiency, emphasising the current lack of empirical research that deeply integrates intervention models with the design of digital games for ASD. This gap hinders the development of truly theory-driven, replicable serious games capable of producing sustainable behavioural change.

To address this gap, this study utilises serious games as a digital medium and the CPS model as its core theoretical framework to construct an integrated intervention model that translates the four stages of CPS into game narrative, task design, and feedback mechanisms. Through a randomised controlled trial, this study examines whether this CPS-integrated intervention yields superior outcomes in terms of social functioning and behavioural integration for children with ASD compared to traditional serious games lacking a clear theoretical framework. The specific research questions are as follows:

(1) How can the CPS model be systematically integrated into the development process of serious games for children with ASD?
(2) Compared with traditional game-based interventions, can serious games incorporating the CPS model more significantly improve the core social functioning of children with ASD? What are the specific manifestations of this improvement?

## 3. Research Methods

### 3.1. General Information

Between March 2024 and June 2025, 48 children with ASD were recruited from a local special education school; their chronological ages ranged from 7 to 12 years (M = 11.40, SD = 1.932). All met the criteria for mild-to-moderate ASD as defined in the ‘ICD-11 Classification and Diagnostic Criteria for Mental and Behavioural Disorders’, and had been placed in high-functioning autism classes by the school’s specialist teaching team following a comprehensive ability assessment. Exclusion criteria: ① confirmed genetic or progressive neurological disorders; ② physical disabilities or other organic impairments; ③ history of head injury or epilepsy; and ④ concurrent conditions requiring pharmacological treatment or other rehabilitative interventions. Exclusion and dropout criteria: ① Inability to continue participation in the intervention due to sudden illness or other reasons; and ② voluntary withdrawal following three consecutive weeks of absence. Stratified randomisation (by year group and baseline ATEC total score) was employed to allocate the children into two groups: the experimental group comprised 32 children, including the cognitive-oriented CPS group (n = 16), the CPS social orientation group (n = 16), and the conventional play control group (n = 16). Two CPS subgroups were established to explore the targeted intervention effects of different generalisation content within CPS games. This study was reviewed and approved by the school’s ethics committee (No. YXLLSP20241002), and the legal guardians or immediate family members of all participants voluntarily signed informed consent forms.

This study is an exploratory pilot trial and no formal a priori power calculations were performed. The sample size of 16 participants per group meets the recommendation for pilot studies that each group should comprise no fewer than 12 participants. To further assess the statistical power of the current sample size for testing the primary intervention effect, this study utilised G*Power 3.1 to conduct a post hoc power analysis. Based on the three-group design of this study (n = 48, α = 0.05), and taking into account the medium-to-large effect sizes (r = 0.39–0.46) observed in the verbal communication and social skills dimensions, the results indicate that the statistical power (1 − β) of the current study is 0.860 > 80%. The current sample size provides preliminary statistical support for the primary outcome measures; however, future findings will require further validation through larger-scale, multi-centre, and long-term follow-up studies.

### 3.2. Method

**Figure 1:**
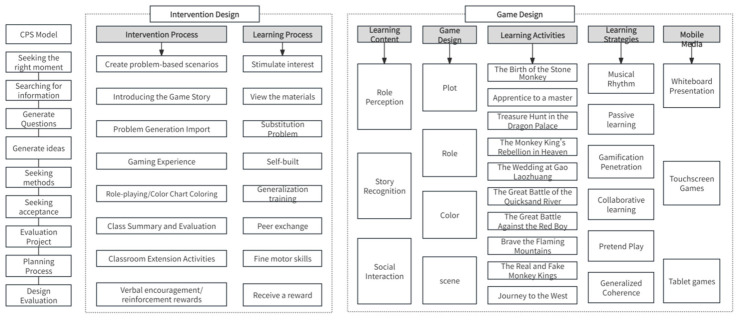
A CPS-Based intervention framework for serious games.

**Figure 2:**
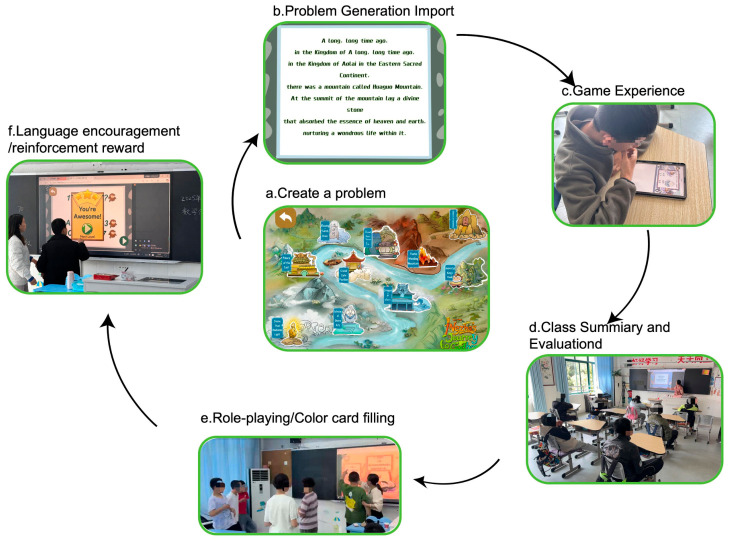
The intervention process.

**Figure 3:**
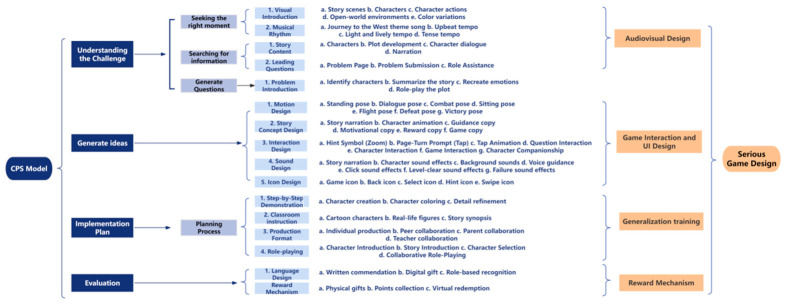
CPS model intervention in the design translation of serious games.

Both groups used serious games for the intervention, with the games sharing the same theme (Ballard et al., 2024). The experimental group adopted the CPS model as its core design framework (see Section 2 for a literature review), systematically translating the four stages of ‘understanding the challenge—generating ideas—seeking acceptance—design and evaluation’ into game narratives, task mechanisms, and feedback systems (see Figure 1, Figure 2 and Figure 3), supplemented by generalisation training such as role-play and artistic creation. The control group utilised a serious game on the same theme, but without the structured guidance of the CPS model or collaborative prompts from teachers; it consisted solely of free exploration and linear task progression. All interventions were conducted in the children’s regular classrooms, with no dropouts during the trial period. The experimental group was subdivided into a cognitive-oriented group and a social-oriented group. Both groups followed the same game structure and process, but differed in the focus of their levels: the cognitive-oriented group focused on levels involving language comprehension and cognitive reasoning, whilst the social-oriented group focused on levels involving social initiation and role-play. The specific distribution of levels is shown in Table 1.

#### 3.2.1. Intervention Objectives

The core objective of this project is to promote the development of children with ASD across the following four dimensions through CPS-driven serious games: ① Verbal communication—enhancing proactive verbal expression and purposeful communicative behaviour; ② social interaction—strengthening interactive skills such as initiating social contact, turn-taking, and collaboration; ③ sensory processing—improving adaptive responses to sensory stimuli; and ④ healthy behaviour—fostering adaptive behaviours such as classroom participation and task persistence. These objectives are progressively achieved through the in-game closed loop of ‘situational immersion → idea generation → collaborative expression → evaluation and feedback’, whilst drawing on the principles of game therapy to promote children’s emotional, social, and cognitive development (Elbeltagi et al., 2023; Liu, 2024; Y.-P. Wang, 2021), as well as the unique advantages of serious games in embedding educational objectives within immersive experiences (Dantas & do Nascimento, 2022; Wilson, 2024; D. Li et al., 2023; Best et al., 2015).

#### 3.2.2. Intervention Framework

This study draws on the CPS model as its theoretical foundation and utilises serious games as a digital intervention medium, proceeding along the following path: firstly, constructing an intervention framework that integrates the CPS model into serious games, thereby translating theory into design; secondly, developing thematic and functional serious game products that take into account the developmental characteristics of children with ASD; thirdly, formulating a structured game-therapy protocol; and, finally, the feasibility of the protocol is validated through multidimensional evaluation.

#### 3.2.3. Intervention Design

Based on the CPS model and design theory, a two-tiered framework comprising ‘pre-design’ and ‘game strategy’ was constructed (see Figure 1). The pre-design layer sequentially maps the four stages of the CPS model to the ten pre-design sessions (Table 1), forming a progressive sequence from ‘basic cognition’ to ‘complex social interaction’; the game strategy layer, meanwhile, translates each stage into specific game elements. Taking the ‘*Sun Wukong’s Adventures*’ series of games—developed based on the Chinese classic *Journey to the West*—as an example, we selected 10 representative chapters: ‘The Birth of the Stone Monkey’, ‘Seeking a Master’, ‘Treasure Hunt in the Dragon Palace’, ‘The Great Uproar in Heaven’, ‘Marriage at Gao Lao Village’, ‘Taking on Disciples at the River of Flowing Sand’, ‘The Great Battle with the Red Boy’, ‘ Brave the Flaming Mountain”, “The True and False Monkey Kings”, and “Journey to the West” for the game design. This study takes *Journey to the West* as the core game narrative material, which has obvious cultural particularity. Differences in participants’ cultural familiarity and story cognition will directly affect their immersion degree, participation enthusiasm, and acceptance of the intervention. Children lacking relevant cultural background may have difficulty understanding game content, resulting in poor intervention engagement. Such cultural context constraints limit the general applicability and popularisation of the proposed intervention scheme. Future research can select more inclusive and culturally universal game themes to eliminate the adverse impact of cultural differences on intervention outcomes. Each level is structured according to the four stages of the CPS model.

Understanding the Challenge Stage: Introducing the problem scenario through visual storytelling. For example, the “Marriage at Gao Lao Village” level uses animation to present the scenario of “villagers seeking help → the need to form a team to rescue them”, requiring children to perceive and define the task objectives (“who are the companions”, “how many people are needed to form a team”) and construct cognitive pathways for “identifying opportunities” and “gathering information”.

Idea Generation Stage: Guiding children to actively generate solutions through interactive choices. For example, the cognitive level “Brave Journey Through the Flaming Mountain” presents a multiple-choice question: “Use the Fan of the Western Paradise or an ordinary fan?”, requiring children to make a judgement based on their prior experience with pattern recognition; the social level “The True and False Monkey King” requires children to identify linguistic clues indicating the true or false characters within dialogue options, with each option triggering a different branch of the storyline.

Seeking Acceptance Stage: Incorporates collaborative role-play and communication tasks. For example, “The Wedding at Gao Lao Village” requires children to take on the roles of Sun Wukong, Tang Sanzang, Zhu Bajie and other characters, completing the task through turn-taking and joint decision-making; cognitive levels practise articulating viewpoints by explaining the reasons for their choices to virtual characters (e.g., “Why did you choose this fan?”).

Design and Evaluation Phase: Immediate, differentiated feedback highlights the consequences of behaviour. For instance, correct choices trigger level-clear animations and reward sound effects, whilst incorrect actions display a hint icon and offer a retry opportunity, encouraging children to reflect and make adaptive adjustments.

Furthermore, based on developmental assessments provided by schools (covering cognition, language, and adaptive behaviour), the game features in-game levels tailored to individual needs (see Figure 2 and Figure 3), ensuring the content is accessible and relevant to daily life. The overall design employs high-contrast colour schemes and a minimalist visual style preferred by children with ASD, with clear and easy-to-understand operational logic.

#### 3.2.4. Intervention Process

Participants were randomised into groups using stratified randomisation: the cognitive-oriented CPS group (n = 16) and the CPS social orientation group (n = 16). Both groups received a serious game intervention incorporating the CPS model; the division into the two subgroups was based on the core training objectives of each level (Table 1). For post hoc exploratory analysis, all children completed all 10 levels; the control group (n = 16) received a conventional game intervention with the same theme but without the guidance of the CPS structure. Both groups aimed to enhance the social functioning of children with ASD (Chen et al., 2023; Guo & Wang, 2024; Miranda et al., 2025), and extraneous variables such as duration, location, and therapist qualifications were strictly controlled throughout the intervention.

The intervention took place in a quiet, comfortable classroom free from distractions. The therapists (four qualified special education teachers, all of whom had completed 8 h of CPS theory and practical training and passed a practical assessment following the training; with the research team conducting monthly spot-check video supervision to ensure consistency in implementation) followed the procedure below: a brief introduction to the game tasks and requirements (2 min); demonstrate the game’s operation using multimedia equipment, whilst assisting the child in interactive activities (15 min); following the demonstration, distribute the game equipment for the child to experience independently (10 min), with the therapist providing no prompts; subsequently, during the generalisation training phase, the therapist assists the child in role-play, art activities, etc. (10 min); and following the intervention, distribute reinforcers (role-play dolls, tokens, colouring cards, etc.) as positive incentives (Weston, 2016). The children’s performance is recorded on video throughout. Once a week, 45 min per session, for a total of 12 weeks. This dosage is based on the school’s standard timetable for mainstream classroom sessions and precedents from previous brief, structured play interventions, and is intended to assess the feasibility of the programme and identify preliminary signals.

#### 3.2.5. Survey of Reinforcing Materials

Reinforcement can enhance the efficiency of behavioural responses and is a crucial and effective factor in shaping behaviour. In this study, drawing on the Child Reinforcer Questionnaire developed by Zhan Fei, a Reinforcer Questionnaire for Children with ASD was devised. Through questionnaire surveys and interviews with teachers, parents, and children to understand the children’s preferences, the researchers found that the participants’ preferred items included: angular-shaped dolls from the game (i.e., merchandise featuring the angular characters from the game), token rewards, and colouring cards.

### 3.3. Evaluation Methods

#### 3.3.1. Behavioural Observation

Classroom activity data for the children was collected via video recording. Each 45 min lesson was divided into five segments: introduction (5 min), games (15 min), independent practice (10 min), generalisation (10 min), and summary (5 min). Two observers, unaware of the group assignments, were assigned to conduct behavioural coding using the BORIS 9.x software at 10 s intervals. Core observation indicators: ① Raising hands to speak, defined as verbal responses or questions initiated proactively without being called upon by the teacher; ② social engagement, defined as initiating eye contact accompanied by verbal or physical interaction, lasting ≥ 2 s; and ③ classroom engagement, etc. A random sample of 30% of the video recordings was coded independently by two observers, and Cohen’s κ coefficient was calculated; if κ < 0.80, retraining was conducted until the threshold was met. A total of 30% of the video footage was extracted in phases during the experiment to assess whether the skill training and transfer demonstrated in the experimental work complied with established standards, and whether the evaluation of participants’ responses was consistent. The inter-observer reliability coefficients (based on percentage agreement) for experimental groups 1 and 2 and the control group were 97.70%, 97.47% and 97.10%, respectively, as shown in Table 2, indicating that the observational data are reliable.

#### 3.3.2. Intervention Fidelity

Six teaching videos were selected at random, and the ‘Intervention Adherence Checklist’ was used to assess whether the intervention process was implemented in accordance with the original plan. This checklist was developed by the research team based on the core components of the CPS, and its content validity was reviewed by two special education experts and two game design experts (item-level content validity index I-CVI ≥ 0.88). It was then independently assessed by two raters using five pre-trial video recordings, yielding an intra-rater reliability coefficient (ICC) of 0.92. Protocol adherence rate = number of steps consistent with the planned steps ÷ total number of steps × 100%. The results showed that the mean protocol adherence rates for the experimental and control groups were 97.77% and 97.22%, respectively, indicating that the trial was strictly implemented according to the planned protocol, as shown in Table 3.

#### 3.3.3. Autism Treatment Evaluation Checklist (ATEC)

Assessments using the ATEC were conducted before and after the intervention, recording scores across four dimensions: language, social interaction, sensory processing, and health behaviour. The ATEC is a widely used self-report scale; although it was applied in numerous ASD intervention studies, its sensitivity to subtle changes and objectivity are inferior to those of blinded clinical assessments (Zhou et al., 2023); the ATEC was selected for this study due to its multidimensional coverage and the fact that it can be completed by teachers in a school setting. Future research should combine it with blinded direct behavioural assessment tools (such as the BOSCC) to enhance measurement validity.

#### 3.3.4. Statistical Analysis

The data were first checked for accuracy, with outliers verified against the original records. Descriptive statistical analysis was performed on the behavioural data. ATEC scores were analysed using SPSS 27.0. The normality of the variances across dimensions was first assessed using the Shapiro–Wilk test. Given the characteristics of this study—namely, its status as a small-sample exploratory intervention study, the presence of significantly non-normal distributions in some variables, and fluctuations in repeated-measure data—this study ultimately adopted non-parametric statistical methods as the primary analytical strategy to enhance the robustness of the statistical results. As most dimensions did not conform to a normal distribution, the Kruskal–Wallis test was used for overall comparisons between groups (reporting H values and *p*-values); where significant overall differences were found, pairwise comparisons were conducted using the Bonferroni-corrected Mann–Whitney U test (cognitive group vs. control group, social group vs. control group, and cognitive group vs. social group), reporting Z-scores, corrected *p*-values, and effect sizes r = Z/√N. Concurrently, a Generalised Estimating Equation (GEE) model was employed to analyse the interaction between group (3 levels) × time (eight measurement points) to test for between-group differences in the rate of improvement. All tests used a two-sided α = 0.05 as the significance level. To improve the transparency of the longitudinal analysis, separate GEE models were fitted for each ATEC outcome domain, with group, time, and the group × time interaction entered as fixed effects. Participant ID was specified as the subject variable, and measurement occasion was specified as the repeated variable. The main effect of interest was the group × time interaction, which tested whether the rate of change differed among the cognitive-oriented CPS group, the social-oriented CPS group, and the control group.

## 4. Research Results

### 4.1. Sample Characteristics and Quality of Intervention Implementation

This study included a total of 48 children with mild-to-moderate ASD, comprising 16 in the cognitive-oriented CPS group, 16 in the CPS social orientation group, and 16 in the conventional play control group. There were no significant differences between the three groups in terms of age (χ^2^ = 1.25, *p* = 0.535), gender composition (χ^2^ = 0.67, *p* = 0.715) or pre-test baseline scores on the various dimensions of the ATEC (all *p* > 0.05), indicating that randomisation was effective and that the groups were comparable.

Assessment of intervention adherence showed that, for the six randomly selected instructional videos, the intervention adherence rates for the experimental and control groups were 97.77% and 97.22%, respectively. Consistency checks for behavioural observation coding revealed that the intra-class correlation coefficients (ICC) for the three observers at each stage were all above 0.96, indicating that the observational data were reliable.

### 4.2. ATEC Scale Results: A Quantitative Assessment of Treatment Outcomes

To determine the suitability of the subsequent statistical analysis methods, this study first conducted normality tests on the relevant continuous variables of the ATEC, including pre-intervention scores, post-intervention scores. Given the relatively small overall sample size in this study (n = 48), with each group comprising 16 participants, the Shapiro–Wilk test was primarily used to assess the normality of the distribution. The results indicated that several variables did not satisfy the assumption of normal distribution.

#### 4.2.1. Pre-Treatment Baseline Equivalence Analysis

To test the comparability of the groups following randomisation, the Kruskal–Wallis H test was used to compare the baseline differences between the three groups prior to intervention. The results showed that there were no significant differences between the three groups in terms of age (H (2) = 0.657, *p* = 0.720), pre-test scores for verbal communication (H (2) = 0.227, *p* = 0.893), pre-intervention social skills (H_2_ = 0.576, *p* = 0.750), pre-intervention sensory processing (H_2_ = 0.281, *p* = 0.869), and pre-intervention health behaviours (H_2_ = 1.283, *p* = 0.527); furthermore, the chi-square test revealed that differences in the gender composition of the three groups were not statistically significant (χ^2^ = 0.000, *p* = 1.000). The above results indicate that the three groups exhibited good baseline consistency and comparability prior to the intervention.

#### 4.2.2. Comparison of Improvements in Prognosis Between Groups

This study employed the Kruskal–Wallis H test to analyse overall intergroup differences in ATEC improvement scores across the three groups. The results showed that, following the 12-week intervention, there were statistically significant differences between the three groups in terms of improvement scores for verbal communication (H (2) = 7.813, *p* = 0.020) and social skills (H (2) = 8.393, *p* = 0.015), suggesting that different intervention approaches yield differences between groups in the effectiveness of improving verbal communication and social skills. In contrast, no significant intergroup differences were observed in the sensory–perceptual improvement scores (H (2) = 2.121, *p* = 0.346), and health behaviour improvement scores (H (2) = 2.025, *p* = 0.363) showed no significant differences between groups, indicating that the improvement effects of different intervention methods in the aforementioned non-core functional dimensions were relatively similar. Table 4 presents the improvement scores, as well as the pre- and post-intervention scores (Mean), standard deviation (SD), and sample size (N).

The gamified intervention based on the CPS model demonstrated more pronounced improvement effects primarily in core social interaction dimensions such as verbal communication and social skills. Consequently, the Mann–Whitney U test was subsequently employed to conduct pairwise comparisons of ATEC improvement scores across groups, aiming to further evaluate the differences in improvement between the various intervention approaches. The results showed that, compared to the control group, the cognitive-oriented CPS group exhibited significantly better improvements in verbal communication (U = 63.00, Z = −2.496, *p* = 0.013, and r = 0.44) and social skills (U = 66.00, Z = −2.351, *p* = 0.019, and r = 0.42). In contrast, improvements in sensory processing and health behaviours were not statistically significant (all *p* > 0.05).

The improvements in language communication (U = 70.00, Z = −2.246, *p* = 0.025, and r = 0.40) and social skills (U = 59.50, Z = −2.605, *p* = 0.009, and r = 0.46) were also significantly superior to those in the control group, with the effect size for the social skills dimension reaching a moderately large level. However, no statistically significant differences were observed between the two groups in the sensory processing and health behaviour dimensions (both *p* > 0.05). Additionally, no significant differences were found between the cognitive-oriented CPS group and the socially oriented CPS group regarding improvements in language communication (U = 116.50, *p* = 0.649), social skills (U = 117.50, *p* = 0.688), sensory processing (U = 113.50, *p* = 0.573), or health behaviours (U = 122.50, *p* = 0.834), indicating high consistency in the overall effectiveness of the two different CPS intervention approaches. These findings demonstrate the following.

Compared to the conventional game control group, both the CPS cognitive-oriented group and the CPS social-oriented group demonstrated significantly better improvements in ATEC’s language communication and social skills, with moderate or greater actual intervention effects. While improvement trends were observed in sensory processing and health behaviours, these differences were not statistically significant.

No statistically significant differences in improvement across all four dimensions were found between the two CPS intervention subgroups. These findings suggest that the overall framework of the CPS model may synergistically promote the concurrent development of cognitive and social abilities (Table 5).

#### 4.2.3. Dynamic Trend Analysis of Repetitive Measurements

The results demonstrated that in the Language Communication dimension, a significant group × time interaction effect was observed (*p* < 0.001), indicating substantial differences in the trajectory of language communication ability improvement across intervention groups over time. Further analysis of ATEC mean score trends at each time point revealed that both cognitive-oriented CPS groups exhibited faster and more stable declines in language communication abilities compared to the control group (a decrease in ATEC scores reflects symptom improvement). Specifically, the average language communication score decreased from 22.62 to 16.38 in the cognitive-oriented CPS group and from 22.56 to 16.56 in the social-oriented CPS group, whereas the control group showed only a decline from 22.31 to 18.13. Similarly, a significant group × time interaction effect was noted in the Social Skills dimension (*p* = 0.009). Dynamic trend analysis indicated that both CPS groups exhibited more pronounced sustained declines during the later stages of intervention compared to the control group. The social-oriented CPS group recorded the largest decrease, with its average social skills score falling from 24.75 to 17.38, while the control group saw only a reduction from 25.12 to 20.56. In contrast, the group × time interaction effects for the sensory processing dimension (*p* = 0.164) and the Health Behaviours dimension (*p* = 0.226) were not statistically significant, indicating that the dynamic improvement trends across different intervention approaches in these non-core functional dimensions showed relatively limited differences.

The GEE results showed a significant group × time interaction for language communication, Wald χ^2^ (2) = 11.613, *p* = 0.003. Compared with the control group, both the cognitive-oriented CPS group (β = −0.296, SE = 0.091, *p* = 0.001, and 95% CI [−0.473, −0.118]) and the social-oriented CPS group (β = −0.258, SE = 0.094, *p* = 0.006, and 95% CI [−0.442, −0.073]) showed faster reductions in language communication scores over time. A significant group × time interaction was also found for social skills, Wald χ^2^ (2) = 11.792, *p* = 0.003. Compared with the control group, the cognitive-oriented CPS group (β = −0.357, SE = 0.130, *p* = 0.006, and 95% CI [−0.611, −0.103]) and the social-oriented CPS group (β = −0.402, SE = 0.120, *p* = 0.001, and 95% CI [−0.636, −0.167]) showed faster reductions in social skills scores over time.

In contrast, the group × time interactions were not significant for sensory processing, Wald χ^2^ (2) = 2.860, *p* = 0.239, or health-related behaviour, Wald χ^2^ (2) = 1.547, *p* = 0.461. These results indicate that the longitudinal trajectories of improvement were clearly differentiated across groups for language communication and social skills, but not for sensory processing or health-related behaviour. Therefore, the longitudinal findings support the stronger effect of the CPS-based serious game intervention on core social-communication outcomes, whereas the effects on sensory processing and health-related behaviour should be interpreted as preliminary and non-significant.

The aforementioned results indicate that the improvement effects of CPS model-based serious game interventions across the core social interaction dimensions are not only statistically significant but also possess substantial practical intervention value. These findings support classifying the current study as exploratory school intervention research.

### 4.3. Behavioural Observation and Social Validity: Practical Verification of Interventional Effects

Specifically, we take the total valid behavioural observation times of each child during one complete intervention period as the unified statistical denominator. All observed classroom behaviours are classified and sorted in advance according to research dimensions, and the final percentage value of each type of behaviour is obtained by dividing the occurrence frequency of a single categorised behaviour by the total observation times within the intervention session. In addition, all participants were grouped strictly in accordance with pre-set experimental grouping standards to ensure the objectivity and accuracy of behavioural percentage statistics. After calculation, the consistency coefficient of each group in each stage was >96%, indicating that the observation results were reliable. Through observation, most children liked and could accept the game intervention experiment and were interested in the role-playing in the game content. A very small number of children, due to individual basic problems, changed from initial resistance to being able to cooperate with teachers to complete tasks by the end of the intervention (Table 6).

#### Social Validity

Social validity is primarily used to determine whether the outcomes of an intervention have significant social implications and bring about changes in individuals’ lives, and it also reflects parents’ and teachers’ satisfaction with the intervention process, methods, and outcomes. Parents and subject teachers expressed their views on the outcomes of the intervention.

One month after the intervention concluded, this study conducted a follow-up survey. Structured questionnaires focusing on children’s daily social adaptive behaviours, interpersonal interaction performance, and intervention acceptance were distributed to caregivers and teachers to collect standardised evaluation data. On this basis, standardised thematic coding will be adopted to sort out all open-ended feedback, and systematic qualitative analysis will be performed to summarise consistent evaluations and practical application effects of the intervention programme.

Parents and homeroom teachers affirmed the intervention outcomes, believing that the game-based intervention significantly improved the social functions of children with ASD, including their cognitive abilities, social skills, and language expression. However, children appeared to prefer the game content of the experimental groups.

The parent of Child A stated that A recognised more characters from *Journey to the West*, could independently draw simple sketches of relevant roles, briefly describe story plots, communicate game stories with classmates, raise hands more actively in class, and express emotions more accurately.

The parent of Child B mentioned that B was unable to draw before the intervention but could colour characters relatively accurately afterward, with noticeable improvements in emotional expression.

The parent of Child C noted that C’s social skills improved significantly, and after the intervention, C actively invited others to join their game activities.

Teachers reported that in the experimental groups, children responded more positively to teachers’ feedback, showed higher enthusiasm for classroom activities, achieved higher accuracy in answering questions, and some participants demonstrated a certain degree of independent learning during tasks, with more frequent sharing of their ideas with others.

## 5. Discussion

The present study examined the effectiveness of integrating the CPS model into serious games for children with ASD. The findings indicate that both CPS-based intervention groups demonstrated significantly greater improvements than the conventional game group in the domains of verbal communication and social skills. In contrast, differences in sensory processing and health behaviours did not reach statistical significance. These results suggest that the CPS framework may be particularly effective in promoting core social-communication abilities, while its influence on broader adaptive functions may require longer intervention duration or additional environmental support.

One notable finding is that improvements in language communication emerged relatively early during the intervention process. Dynamic trend analysis showed that the language communication scores of the CPS groups declined more rapidly and steadily than those of the control group. This outcome may be related to the structured interaction logic embedded within the CPS framework. Unlike conventional game interventions that primarily emphasise task completion, the CPS-based games guided children through sequential stages of identifying problems, generating responses, expressing choices, and receiving feedback. Such a process created repeated opportunities for purposeful verbal expression within meaningful contexts. For children with ASD, whose communication difficulties are often associated with reduced social initiation and limited communicative motivation, structured interactive scenarios may provide clearer communicative cues and lower the cognitive burden associated with spontaneous expression.

The improvements observed in social skills further support the intervention value of CPS-oriented game design (Tang et al., 2023). In the present study, many game tasks required collaborative participation, turn-taking, role negotiation, and joint decision-making. Behavioural observation data showed that, during the later stages of the intervention, the proportion of children actively participating in classroom interaction increased substantially, while passive or non-engaged behaviours decreased markedly. These changes indicate that the CPS framework may facilitate social participation by transforming abstract social rules into concrete and operable problem-solving processes. Previous studies have suggested that children with ASD tend to perform more effectively in predictable and highly structured environments. The CPS model appears to align with this characteristic by providing explicit procedural scaffolding during social interaction, thereby reducing uncertainty and enhancing participation willingness.

Another important finding is that no statistically significant differences were found between the cognitive-oriented CPS subgroup and the social-oriented CPS subgroup across all ATEC dimensions. This result suggests that the intervention effects of the CPS framework may not depend solely on the thematic orientation of individual game tasks. Rather, the structured problem-solving process itself may function as the primary mechanism underlying behavioural improvement. Although the two CPS subgroups differed in task emphasis, both incorporated the same sequential stages of challenge recognition, idea generation, collaborative interaction, and evaluative feedback. This shared structural logic may have promoted the parallel development of communication, cooperation, and adaptive participation (Greene et al., 2003).

In the dimensions of sensory processing and health behaviour, the CPS groups demonstrated improvement trends compared with the control group, although these differences did not reach statistical significance. Several factors may account for this outcome. First, sensory adaptation and behavioural regulation are often influenced by long-term environmental experiences and daily routines, making short-term changes more difficult to detect. Second, the intervention frequency in this study was relatively limited, consisting of one session per week over twelve weeks. While such intensity may be sufficient to influence communication-related behaviours, it may not be adequate to produce stable changes in broader adaptive functioning. From the perspective of intervention dosage, more entrenched adaptive functions—such as sensory adaptation and health behaviours—may require a substantially higher cumulative intervention dose to yield significant improvements, as these functions are deeply embedded in children’s daily routines, and the dose required for their reshaping may considerably exceed the parameters of the current design. Nevertheless, observational records and parent feedback indicated that some children demonstrated increased classroom engagement, improved emotional expression, and greater willingness to participate in daily activities, suggesting potential practical value beyond the statistical findings.

The social validity results further support the feasibility and acceptability of the intervention. Parents and teachers generally reported positive changes in children’s communication willingness, classroom participation, emotional expression, and peer interaction. In particular, some children began to initiate interaction more actively or demonstrated increased enthusiasm toward collaborative activities. These observations suggest that CPS-based serious games may possess ecological relevance beyond the experimental setting. Compared with highly repetitive training paradigms, narrative-driven and role-based interactive experiences may be more easily accepted by children with ASD and may contribute to sustained participation over time.

Apart from the core intervention effect, other potential influencing factors should not be ignored. The novelty effect of game activities and improved individual participation motivation may all contribute to children’s behavioural improvements. These confounding variables cannot be completely excluded in this study, which should be noted in result interpretation. Although the behavioural observation coding in this study was conducted by raters who were unaware of group assignment and achieved high inter-rater reliability (ICC), the study design did not incorporate blinded standardised clinical assessment tools. Consequently, our findings still have certain limitations and should be interpreted as school-based preliminary evidence rather than definitive clinical conclusions. Future studies are encouraged to incorporate blinded clinical or direct behavioural assessments—such as the ADOS-2, SRS-2, or BOSCC—to capture treatment-induced behavioural changes more comprehensively and objectively. In addition, the comparison between the cognitive-oriented and social-oriented CPS groups was treated as exploratory. No significant differences were found between the two CPS subgroups across language communication, social skills, sensory processing, or health-related behaviour, suggesting that the observed benefits may be more closely related to the overall CPS-structured intervention process than to clearly differentiated related pathways.

The relatively small sample size of this study may limit the generalizability and external validity of the findings to some extent. This is because the study was conducted at a single special education school with a sample size of only 48 participants, who were evenly allocated across three groups. For a three-arm repeated-measures intervention study involving multiple outcome measures, this sample size remains modest. This limitation largely stems from the fact that the intervention had to be embedded within the school’s regular classroom schedule and was carried out under the premise of maintaining normal teaching order, which inevitably constrained participant recruitment and data collection intensity. Nevertheless, post hoc power analysis indicated that the current sample provided sufficient statistical power (1 − β = 0.86) to detect the primary effects in core domains, which offers preliminary support for the main findings. Therefore, the results of this study should be interpreted as preliminary and exploratory. Notably, we observed statistically significant improvements in language communication and social skills among children with ASD. These findings provide empirically valuable signals for the design of CPS-based serious games. However, their robustness should be further verified through larger-scale, multi-centre studies employing a priori power calculations, longer follow-up periods, and more sensitive blinded behavioural or clinical assessments. Furthermore, it should be acknowledged that factors beyond the CPS framework may have also contributed to the observed improvements and cannot be completely excluded. However, these general factors would likely have affected all groups comparably and thus cannot fully account for the differential improvements observed specifically in the CPS groups’ core social-communication outcomes. What distinguishes the CPS intervention is its structured, process-oriented logic that systematically scaffolds problem-solving, communication, and collaboration, aligning with behavioural science principles of scaffolded skill building to foster sustainable engagement. Therefore, the primary contribution of this study is not to assert the superiority of a specific “CPS therapy,” but to provide a proof-of-concept for systematically translating a structured educational model into serious game mechanics to support theory-driven digital intervention design for children with ASD.

Despite these limitations, the present study provides preliminary empirical support for integrating structured educational models into serious game interventions for children with ASD. Rather than treating games solely as motivational tools, this study emphasises the importance of aligning game mechanics with coherent intervention logic. The CPS framework offers a process-oriented pathway through which serious games may support active participation, social communication, and collaborative engagement. This design approach may contribute to the development of more theory-driven and replicable digital intervention programmes within special education contexts.

## 6. Conclusions

This study explored the application of the CPS model in serious games designed for children with ASD and evaluated its intervention effectiveness through a 12-week randomised controlled trial. The findings indicate that CPS-based serious games produced significantly greater improvements than conventional game interventions in the domains of verbal communication and social skills. Behavioural observation and social validity data further demonstrated increased classroom participation, greater interaction willingness, and improved collaborative engagement among children in the CPS groups.

The results suggest that integrating the four stages of the CPS model into game narratives, task structures, and feedback mechanisms may enhance the coherence and intervention value of serious games. By transforming social interaction and communication tasks into structured problem-solving experiences, the CPS framework appears to provide children with ASD with clearer behavioural guidance and more accessible opportunities for active participation.

Although the intervention effects in sensory processing and health behaviour did not reach statistical significance, positive developmental trends were still observed. These findings indicate that CPS-based serious games may have broader rehabilitative potential, while also highlighting the need for longer intervention periods and more comprehensive assessment strategies in future research.

Overall, this study provides preliminary evidence that theoretically structured serious games can support the social functioning of children with ASD. The integration of educational theory, digital design, and behavioural intervention may offer a promising direction for the future development of customised and evidence-based digital interventions within special education settings.

## Figures and Tables

**Table 1:** Serious games and intervention contents.

Serial Number	Game Name	Theme	Type	Generalisation Category	Intervention Content
1	Birth of the Stone Monkey	Role Cognition	Cognitive	Role-Playing	Role-playing as Sun Wukong
2	Learning Arts from a Master	Colour Cognition	Cognitive	Role Drawing	Role-playing as Sun Wukong and Patriarch Bodhi
3	Seeking Treasures in the Dragon Palace	Number Cognition	Social	Electronic Game	Digital Calculation
4	Uproar in Heaven	Graphic Cognition	Cognitive	Electronic Game	Graphic Discrimination
5	Marrying the Bride at Gao Village	Cooperation and Mutual Assistance	Social	Role-Playing	Role-playing as Sun Wukong, Tang Seng, and Zhu Bajie
6	Battle at Flowing Sand River	Role Cognition	Social	Role-Playing	Role-playing as Sun Wukong, Tang Seng, Zhu Bajie, and Sha Wujing
7	Battle with Red Boy	Colour Cognition	Cognitive	Role Drawing	Role-playing as Sun Wukong, Tang Seng, Zhu Bajie, and Red Boy
8	Braving the Flaming Mountain	Geometric Cognition	Cognitive	Electronic Game	Geometric Shapes
9	True and False Sun Wukong	Joint Collaboration	Social	Role-Playing	Role-playing as Sun Wukong, Zhu Bajie, Sha Wujing, and Six-Eared Macaque
10	Obtaining the Scriptures from the West	Story Cognition	Social	Role-Playing	Role-playing as Sun Wukong, Tang Seng, Tathagata Buddha, and Guanyin Bodhisattva

**Table 2:** Observer consistency scoring table.

Subject Group	Pretest	Intervention	Post-Test	Overall Average
Fidelity % (Experimental Group 1)	96.6	98.20	98.40	97.70
Fidelity % (Experimental Group 2)	97.00	97.40	98.00	97.47
Fidelity % (Control Group)	96.4	97.20	97.6	97.10

**Table 3:** Intervention fidelity detection form.

Classroom Video	Planned Steps (Experimental Group)	Actual Steps (Experimental Group)	Fidelity % (Experimental Group)	Planned Steps (Control Group)	Actual Steps (Control Group)	Fidelity % (Control Group)
1	14	13	92.86	12	11	91.67
2	12	12	100.00	10	10	100.00
3	16	15	93.75	12	11	91.67
4	12	12	100.00	10	10	100.00
5	12	12	100.00	10	10	100.00
6	14	14	100.00	12	12	100.00
Average	—	—	97.77	—	—	97.22

**Table 4:** Descriptive statistical results (Mean ± SD) for each dimension of the ATEC across the three groups.

Variable		Cognition-Oriented CPS Group (n = 16)	Socially Oriented CPS Group (n = 16)	Control Group (n = 16)
Verbal communication	Pre-test	22.63 ± 2.50	22.56 ± 2.56	22.31 ± 2.39
	Post-test	16.38 ± 3.07	16.56 ± 2.76	18.13 ± 3.79
Social skill	Pre-test	25.31 ± 2.85	24.75 ± 2.65	25.13 ± 2.45
	Post-test	18.25 ± 4.06	17.38 ± 3.61	20.56 ± 4.41
Sensory processing	Pre-test	23.00 ± 3.69	22.38 ± 3.86	23.00 ± 2.85
	Post-test	17.88 ± 4.26	17.50 ± 4.26	18.88 ± 4.35
Health behaviour	Pre-test	12.63 ± 3.26	12.38 ± 3.03	11.31 ± 1.89
	Post-test	7.81 ± 2.69	7.44 ± 1.26	7.63 ± 2.42
Language communication improvement score		−6.25 ± 1.61	−6.00 ± 1.75	−4.19 ± 2.07
Social skill improvement score		−7.06 ± 2.35	−7.38 ± 1.86	−4.56 ± 2.92
Perceptual improvement score		−5.13 ± 1.20	−4.88 ± 1.15	−4.13 ± 2.13
Health behaviour improvement score		−4.81 ± 4.39	−4.94 ± 2.91	−3.69 ± 3.20

Note: Data are expressed as Mean ± SD. The improvement score is calculated as “post-intervention score–pre-intervention score”; more negative values indicate greater symptom improvement.

**Table 5:** Pairwise comparison results of ATEC improvement scores (Mann–Whitney U test results for improvement scores).

Comparison Group	ATEC Dimension	U Price	Z Price	*p* Value	Effect Size r	Result
Cognition-oriented CPS group vs. control group	Verbal communication	63.00	−2.496	0.013	0.44	Significant
	Social skill	66.00	−2.351	0.019	0.42	Significant
	Sensory processing	92.50	−1.357	0.175	0.24	Not significant
	Health behaviour	101.00	−1.026	0.305	0.18	Not significant
Socially oriented CPS group vs. control group	Verbal communication	70.00	−2.246	0.025	0.40	Significant
	Social skill	59.50	−2.605	0.009	0.46	Significant
	Sensory processing	102.00	−0.996	0.319	0.18	Not significant
	Health behaviour	91.50	−1.387	0.165	0.25	Not significant
Cognition-oriented CPS group vs. social-oriented CPS group	Verbal communication	116.50	−0.455	0.649	0.08	Not significant
	Social skill	117.50	−0.401	0.688	0.07	Not significant
	Sensory processing	113.50	−0.564	0.573	0.10	Not significant
	Health behaviour	122.50	−0.210	0.834	0.04	Not significant

Note: The improvement score is calculated as “post-intervention score–pre-intervention score,” with more negative values indicating greater symptom improvement. The effect size (r) is calculated using the formula r = |Z|/√N. According to Cohen’s (1988) criteria, an r = 0.1 represents a small effect, r = 0.3 a moderate effect, and r ≥ 0.5 a large effect.

**Table 6:** Changes in observed classroom engagement behaviours from early to late intervention.

Observation Content	Category	Early Stage	Late Stage
Raising Hands to Speak	No Speech	60%	10%
	1~2 Times	30%	50%
	>2 Times	10%	40%
Social Interaction	No Participation	55%	10%
	1~2 Times	30%	20%
	>2 Times	15%	70%
Health Behaviours	No Behaviour	45%	10%
	1~2 Times	40%	55%
	>2 Times	15%	35%

## Data Availability

The datasets presented in this article are not readily available because sharing our dataset would lead to personal privacy, moral, and ethical issues. Requests to access the datasets should be directed to ysxyshl@ahut.edu.cn.
